# TreC_Metha: A Digital Application to Enhance Patient Agency, Therapy Compliance and Quality of Life in Metastatic Breast Cancer Patients

**DOI:** 10.3390/curroncol32060299

**Published:** 2025-05-23

**Authors:** Antonella Ferro, Maria Chiara Pavesi, Lucia Pederiva, Claudio Eccher

**Affiliations:** 1 Medical Oncology and Rete Clinica Senologica, Azienda Provinciale per i Servizi Sanitari, 38123 Trento, Italy; 2 Operating Unit of Psychology and Rete Clinica Senologica, Azienda Provinciale per i Servizi Sanitari, 38123 Trento, Italy; mariachiara.pavesi@apss.tn.it; 3 Center for Digital Health, Fondazione Bruno Kessler, 38123 Trento, Italy; lpederiva@fbk.eu (L.P.); cleccher@fbk.eu (C.E.)

**Keywords:** digital health, mobile application, breast cancer, metastatic breast cancer, digital therapeutics, patient empowerment, informed decision-making, healthcare, quality of life

## Abstract

The prognosis for Hormonal Receptor positive-HER2-negative (HR+ HER2-negative) metastatic breast cancer (mBC) has significantly improved by advances in hormone therapies, targeted drugs, and antibody–drug conjugates (ADCs). Nevertheless, maintaining quality of life (QoL), managing symptoms, and reducing treatment-related toxicity remain essential. Background: eHealth solutions offer new opportunities to enhance patient engagement and well-being through digital tools. This paper aims to delineate the fundamental functionalities and objectives of TreC_Metha, a technologically advanced instrument to provide effective support during all care process of patients diagnosed with HR+HER2-negative mBC able to proactively change its configuration depending on the treatment line or on the intra-line treatment phase the patient undergoes, as set by the healthcare team. Methods: The TreC_Metha platform was developed through a structured, evidence-based four-phase process aimed at scalability, usability, and clinical relevance. The development began with a formal analysis of the metastatic breast cancer (mBC) care pathway using BPMN modeling to map phases, activities, and stakeholders, highlighting differences from early-stage breast cancer. This analysis informed the identification of key points where digital support could enhance care. Patient needs were assessed through a web-based questionnaire (N = 20) and two focus groups (N = 11), enabling a participatory design approach. Based on these insights, the platform’s functional and non-functional requirements were defined, leading to the design and implementation of a patient-facing mobile app and a clinical dashboard tailored to mBC-specific needs. Results: Preliminary findings from the web survey focus groups revealed significant gaps in communication and information delivery during the mBC care journey, contributing to patient anxiety and reduced confidence. Participants expressed a preference for digital and printed resources to improve understanding and facilitate interactions with healthcare providers. These insights informed the development of the TreC_Metha platform. The clinical dashboard enables real-time monitoring and decision-making, while the mobile app supports bidirectional communication, therapy adherence, and patient-reported data collection. A system prototype is currently under refinement and will undergo usability testing with a small cohort of users. Following this phase, the pilot study will evaluate the platform’s impact on QoL, aiming for a ≥10% improvement in outcome measures and contributing to a more patient-centered care model in the mBC setting. Conclusions: TreC_Metha represents an innovative tool that may enable involvement and active participation in the mBC care process for both a multidisciplinary care team of professionals and the patient, and that can be easily adapted to other cancer types and chronic diseases.

## 1. Introduction

### 1.1. Background

Metastatic breast cancer (mBC) is a debilitating disease that remains incurable and presents a significant societal, medical, and economic burden worldwide, causing over 620,000 deaths in 2018, despite treatment improvements [1]. It has been observed that mBCs may present as either “de novo” disease, characterized by the occurrence of distant metastases at the time of diagnosis, accounting for 4.1–8.5% of cases, or as recurrent mBC in patients (up to 30%) who have previously undergone definitive therapy for early breast cancer (BC) [2,3,4]. The advent of novel treatment modalities has resulted in prolonged survival rates. The median overall survival (OS) for mBC is approximately three to five years, with this being influenced by a variety of factors, including patient characteristics (e.g., age, concomitant pathologies, performance status, general conditions), disease burden, BC subtypes (luminal A and B, HER2 and triple negative), metastases sites and access to treatment [5]. Recent data have indicated a trend of OS improvement in specific subtypes (luminal A and B, and HER2-positive) of mBC with target therapies that exceed a five-year survival rate [6]. Typically, mBC involves a prolonged sequence of treatments, characterized by periods where the disease is under control but then relapses, necessitating modifications or changes in therapeutic strategies. While improving disease control and overall survival is paramount, these advances must go hand in hand with maintaining or improving quality of life (QoL), effectively managing symptoms, and minimizing treatment-related toxicity. Prolonged treatments for metastatic disease have been demonstrated to intensify emotional issues and psychosocial distress, a condition which the majority of patients diagnosed with BC experience to varying degrees [7]. For some individuals, distress may diminish over time; however, for others, the ongoing uncertainty, physical burden, and emotional toll can severely compromise psychological well-being. Patients may experience adverse psychological side effects such as depression, anxiety, and fatigue, or social side effects such as reduced ability to work, strained family dynamics, and difficulties in intimate relationships [8,9,10,11,12,13,14,15,16,17]. It has been demonstrated that patients who perceive themselves as active participants report a reduced number of health concerns, a greater sense of perceived control, and improved health outcomes [18]. This active orientation has been found to correlate with superior disease management, improved functional capacity, and enhanced treatment adherence. In this context, patient-reported outcome measures (PROMs) have been identified as a potentially valuable addition to the management of mBC, facilitating the monitoring of critical symptoms and adverse effects, thereby helping to prevent severe cases that require emergency care. Additionally, they can assist in identifying patients who require additional medical intervention and in the more effective management of patients’ medical needs [19].

### 1.2. Role of Digital Tools in Breast Cancer and Previous Works

The advent of eHealth solutions has precipitated the integration of technology into the management of mBC, thereby facilitating the delivery of digital content and promoting patient engagement in initiatives designed to enhance their physical and emotional well-being. It has been documented that the utilization of electronic applications for the purpose of Patient-Reported Symptom Monitoring has the potential to enhance QoL [20,21,22] and improve long-term outcomes [23,24,25,26,27,28]. The preponderance of research findings indicates that digital health platforms have the potential to enhance patient outcomes and quality of life by means of increasing patient education, encouraging patient involvement, promoting treatment adherence, and reducing illness anxiety [29].

The CAMA application was developed to assist in the self-management of patients with breast cancer and survivors of cancer, enabling them to manage their condition independently. The platform offered customized, evidence-based information from clinicians, medication and appointment trackers, and an asynchronous question-and-answer feature with healthcare professionals. A study of 72 participants demonstrated that the utilization of CAMA resulted in a significant enhancement in self-efficacy, anxiety, and depression levels [30]. In the randomized controlled trial conducted by Handa et al., the efficacy of the BPSS app in providing support to BC patients undergoing chemotherapy was evaluated. While the application did not demonstrate a substantial improvement in anxiety, depression, or health literacy, it enhanced communication between patients and medical staff by revealing discrepancies in the assessment of severe side effects [31].

The mPRO Mamma study indicated that using a mobile application for symptom tracking and tailored support significantly improved the quality of life and functional well-being of early-stage breast cancer patients receiving systemic therapy. Notably, the study found no change in the use of health resources between the groups that used the app and those that did not [32]. A Chinese multicentre randomized controlled trial, the e-Support study, assessed a mobile app for BC patients undergoing chemotherapy. The app incorporated educational materials, expert Q and A, peer support, and personal narratives. Over 12 weeks, app users showed significant improvements in self-efficacy, reduced symptom interference, and better quality of life compared to standard care. However, these benefits did not persist at the 6-month follow-up [33]. The study by Mohammadzadeh et al. [34] focused on the creation and assessment of an Android application designed to help Iranian women manage their BC. Over a period of three months, participants demonstrated substantial improvements in various domains of quality of life, including management, reduced negative feelings, and increased social engagement. The application attained an exceptional usability rating of 83 out of 100, indicating its effectiveness and ease of use. In 2019, Buscemi et al. presented a feasibility study of My Guide, a culturally adapted smartphone application for Hispanic breast cancer survivors. During the course of a four-week period, the participants demonstrated elevated levels of engagement, satisfaction, and enhanced cancer-related knowledge. The results obtained from the study confirm the acceptability of the application and its potential for wider implementation [35]. The same authors then (2020) evaluated the use of 2 culturally informed, evidence-based smartphone apps for Latina breast cancer survivors—one that was designed to improve HRQoL and reduce symptom burden (My Guide) and the other to promote healthy lifestyle behaviors (MyHealth). Clinically meaningful improvements were observed in BC well-being among low app users (i.e., <60 min of use/week) of My Guide and social well-being among high app users (i.e., ≥60 min of use/week) of My Health [36]. The Pink Journey app, developed and tested by Fang et al. [37], aimed to support women deciding on breast reconstruction after mastectomy. Incorporating shared decision-making and values clarification, a study with 11 participants found the app user-friendly and effective in reducing decisional conflict, improving understanding of surgical options, and fostering more confident decisions about breast reconstruction. Ponder et al. [38]. conducted a feasibility study on a mobile health application designed for patients undergoing breast cancer surgery. The application’s objective was to provide support to patients by means of symptom tracking, educational resources, and communication tools. A 2021 observational study by Yu et al. investigated the effect of a smartphone application on how well early-stage breast cancer patients followed multidisciplinary treatment (MDT) advice. Examining data from 4475 patients, the study found that those who used a mobile health (mHealth) app showed notably better adherence, especially concerning adjuvant chemotherapy and radiotherapy [39].

Most of the digital tools described above typically have a narrow focus and provide a limited number of functions, such as information provision, adherence support, lifestyle advice, psychological assistance, or resource referral. These applications are often not designed to offer a comprehensive, integrated platform. Furthermore, a significant number of these systems are not equipped with the capacity to generate personalized recommendations that are informed by the unique data of individual patients. A further challenge frequently encountered pertains to the concept of usability, a problem that is especially pertinent when considering the diversity of user groups.

Furthermore, the majority of interventions and applications are developed with minimal to no input from the end-user. The effectiveness of such interventions can be considerably enhanced by employing co-design tools, which are defined as a participative approach that involves end-users and other relevant stakeholders in all aspects of intervention development. This participative process encompasses the assessment of needs, the development of content, the testing of pilot versions, and the completion of the final model [40,41].

What differentiates TreCMetha from most of the applications mentioned above is that it is conceived as a comprehensive system designed to manage the entire mBC care pathway. It provides support to both patients and healthcare professionals, enabling the latter to continuously monitor the patient’s status and requests, and to intervene promptly when necessary.

### 1.3. Study Aim

In this context, we designed the TreC_Metha project, a digital platform aimed at supporting patients undergoing treatment for luminal HR+/HER2-negative mBC. This platform aims to facilitate the exchange of information between patients and healthcare teams, provide guidance on organizational aspects and clinical stages of the healthcare process, and promote greater patient awareness and engagement in their care journey. By fostering informed decision-making, TreC_Metha contributes to improving both the physical and psychosocial well-being of patients. Beyond individual patient benefits, the platform has the potential to enhance clinical monitoring, patient-care team relationships, and interdisciplinary collaboration among healthcare professionals. This can further improve integration, both within hospital settings and between hospitals and community healthcare services, contributing to the sustainability of the healthcare system by reducing unplanned and unnecessary hospital admissions, shortening hospital stays, and minimizing patient transport to hospitals. The advantage of Trec_Metha is that it is designed for the entire process of care of patients with MBC and not just for a specific context. In fact, the application layers of our platform include the following:

- Home therapy administration diaries;
- Monitoring of drug toxicities and disease-related symptoms;
- Systems to improve adherence (through alerts and/or educational support);
- Support for mental health aspects;
- Information on symptoms of disease and on collateral effects of its therapies;
- Educational support on promoting and improving lifestyles;
- Administering and gathering questionnaires to assess communication comprehension, well-being, satisfaction, and quality of life;
- Use of a chat between doctor/healthcare personnel and patient;
- Recording personal narratives.

Furthermore, integration with the Health Care Service Trust’s information systems facilitates seamless interconnection with both clinical and organizational data, such as scheduled appointment information.

Finally, another peculiarity of our project is that it has been designed in strict collaboration with the final users through participatory design sessions involving the various stakeholders through interviews or focus groups. The objectives of the focus groups comprised the collection of patients’ experiences, as well as the identification of their primary needs, difficulties, and information gaps perceived during disease pathways.

We believe that this approach can lead to the development of a more comprehensive and culturally appropriate tool, unlike similar applications, which can address the multiple needs of individuals living with MBC.

The TreC_Metha project is founded on an innovative approach that integrates these objectives into a cohesive system. It is hypothesized that the application will benefit patients, healthcare providers, and organizations alike, as well as ensure a more effective and patient-centered treatment pathway, as reported in Table 1. In this paper, we present the analysis phase of the mBC care process, along with the objectives and functionalities that a technological tool like the TreC_Metha platform must have to effectively support the HR+HER2 negative mBC care pathway. In the next steps, a fully functional prototype that provides a realistic representation of its visual structure and functionality will be tested on a limited number of patients. Only after this step, a pilot study will evaluate the acceptability, usability, and impact of TreC_Metha on QoL of 50 HR+HER2-negative patients. The sustainability and the expected outcomes of the project approach will be assessed through a dedicated analysis that will consider the following:

1. The perceived value of the overall solution following a value-based care approach by: disease awareness questionnaire before and after use of the platform; distress thermometer use; final satisfaction questionnaire; QoL measures questionnaire;
2. The cost–benefit outcomes of implementing this solution in terms of number of people that could benefit against the overall cost (e.g., reduction in unplanned and unnecessary admissions to hospital, in general, and to the emergency department in particular; reduction in hospital admissions or their duration due to treatment side effects; reduction in patient transport at hospital expense).

The paper is organized as follows: Section 2 outlines the phases of the platform’s design and development process. Section 3 presents the results, including the survey and focus group findings, the analysis of the clinical process, the technological solution, and the system’s structure and functionalities. Finally, Section 4 and Section 5 provide the discussion and conclusions, respectively.

**Table 1:** Specific objectives of the TreC_Metha project.

Objectives for Patients	
Education	Provision of training, educational resources, and motivational support during periods of treatment, as well as during therapeutic breaks.
Patient Empowerment	The utilization of a diary has been identified as a method to stimulate active involvement in healthcare and decision-making, as well as the incorporation of patient-reported outcome measures (PROMs) and patient-reported experience measures (PREMs).
QoL Measurement	The utilization of PROMs and PREMs encompassing QoL scales allows for the evaluation of physical and mental health.
Objectives for the Healthcare Team	
Data Utilization	Collection and use of patient-generated health data (PGHD), such as self-reported side effects, to adjust treatment plans.
Improved Communication	Improvement of communication and co-operation between healthcare providers.
Intervention Tracking	The maintenance of comprehensive records of interventions during the course of the care process.
Objectives for healthcare organizations	
Reduction in unplanned and unnecessary admissions to the hospital, in general, and to the emergency department in particular; Reduction in hospital admissions or their duration due to treatment side effects (e.g., toxicity); Reduction in patient transport at hospital expense	

## 2. Materials and Methods

The design of the TreC_Metha application is based on a robust and systematic development framework; this ensures efficiency, user-friendliness, and scalability.

Its evidence-based methodology prioritizes iterative development through continuous feedback and alignment with clinical goals, ensuring that the proposed solution is both effective and tailored to end-user needs.

The proposed digital tool relies on the TreC platform’s microservice-based middle layer [42], which enables the management of personal and clinical data and provides services to empower patients, with functionalities tailored to the specific mBC domain. The platform ensures privacy-by-design compliance with the GDPR (General Data Protection Regulation).

The entire design and development process was divided into four main phases. While this paper focuses solely on presenting the analysis activities and the functionalities of the technological tool, the following section outlines all the phases.

Phase 1: Analysis of the metastatic BC care path and determination of system requirements.

A detailed analysis of the phases, activities, and actors involved in the mBC care path was conducted to identify differences from early BC and represented using formal process notation BPMN (Business Process Modeling and Notation (Object Management Group, Business Process Model And Notation (BPMN) https://www.omg.org/spec/BPMN/2.0; accessed on 1 December 2010)), an OMG (Object Management Group) standard that combines a graphical notation for the formal and unambiguous representation of procedures with the ability to communicate in a way that is understandable to individuals who are not familiar with formal languages. This analysis helped determine at which stages the TreC_Metha platform can support patients and healthcare professionals. The Trec_Metha app for the patients has been designed in strict collaboration with the final users through participatory design sessions involving the various stakeholders through interviews or focus groups. In the initial phase of participatory design, the distribution of a questionnaire was conducted through the web (CAWI) to a cohort of patients (N = 20) with the aim of assessing the level of awareness concerning their pathway, determining the extent to which their information needs were met, and identifying the necessary measures to address the prevailing information deficit. Following survey analysis, two focus groups were conducted on two separate occasions, with a total of 11 patients (of differing ages, social backgrounds, and educational qualifications). The facilitation of these groups was overseen by a facilitator with extensive experience in the field of user experience.

Phase 2: Metha system design and implementation

Based on the needs and system requirements identified in Phase 1, Phase 2 focuses on defining the system’s functional and non-functional requirements and designing and implementing the backend services, the dashboard for the healthcare professionals, and the app for patients, necessary to implement the functionalities determined in Phase 1 for the TreC_Metha platform [43]. Starting from the instance of the TreC Arianna platform, which was developed to support the early breast cancer process (see Section 3), the system has been adapted by modifying existing components and developing new modules to better address specific needs. Specifically, the system integrates specific personal observations, diary, drug toxicities, disease-related symptoms tracking, educational support material, and a clinical dashboard.

Phase 3: Creation and integration of the material delivered by the app

The objective of this phase is the collection, creation, and adaptation of information and documents, as well as the delivery of tutorials to patients via the Trec_Metha app. The material provided is selected on the basis of an analysis of patient needs and then structured in accordance with the principles of empathic communication. It is organized into three main elements: normalization and validation, education, and the sharing of strategies to give patients a sense of control and agency in their treatment path. The material was designed to be delivered through a chatbot, which will be developed in a subsequent phase to monitor the patients’ understanding of the communications received from clinicians and their adherence to the mBC treatment. This approach ensures that the material itself could serve as a source of emotional support for the patients by providing tailored support, facilitating health data collection, and promoting bidirectional communication. Simultaneously, it empowers patients by asking them to take an active role in their own treatment. This interactive engagement allows patients to process and cope with the treatment regimens while fostering a deeper understanding of their treatment.

Phase 4: Feasibility Pilot Preparation and Execution

This phase is intended to define and organize a pilot qualitative study that has already been approved by an ethical committee. The study will span a minimum of four months and a maximum of six months. During this period, patients will input data about side effects of the therapy, general condition, performance status, and, in general, all the relevant parameters related to the mBC pathway, with the target of improving at least 10% score measured by pre-post dedicated questionnaires (such as mood, anxiety, perceived quality of life—BR45, HRQoL, QLQ-ELD14, and EORTC QLQ-C30 [44,45,46]). This information will be timely presented to clinical professionals through the web dashboard and will support decision-making during the treatment process. All these parameters will be taken into account for assessing the feasibility of the system in terms of patient-reported experience (PREMS), patient-reported outcomes (PROMS), and clinicians’ expectations.

The objective of this data collection is to assess the acceptability and feasibility of the platform (primary objective of the trial). Furthermore, data pertaining to psychological and perceived QoL parameters will be collected, with the intention of facilitating comprehension of QoL aspects (secondary objective of the trial). The initial assessment will also provide a baseline reference to oriented treatment and to better understand patient needs. The data will also be used to promote a more patient-need-orientation care pathway, especially for the psychological aspect.

**Figure 1:**
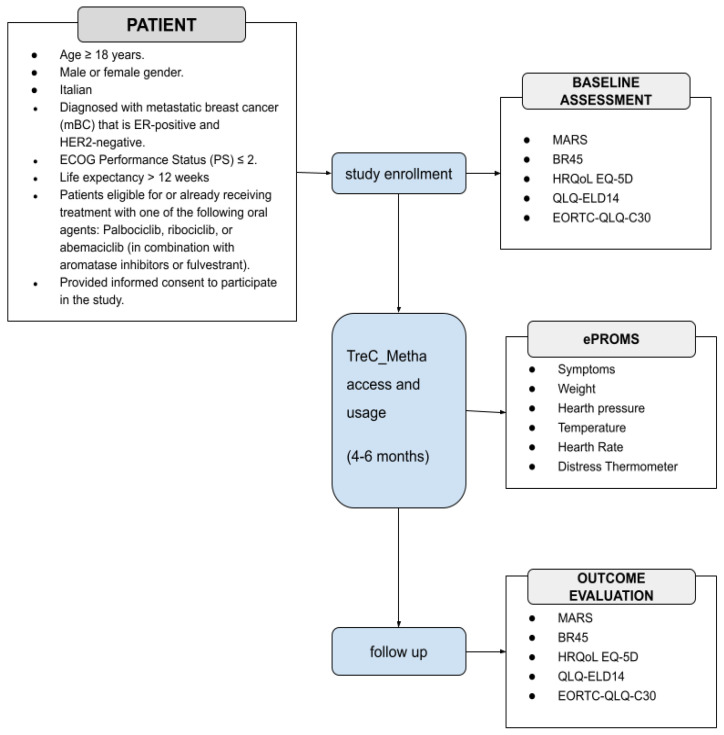
Schematic representation of the pilot study. ECOG: Eastern Cooperative Oncology Group; MARS User Mobile Application Rating Scale; BR45 European Organisation for Research and Treatment of Cancer Quality of Life Questionnaire updated breast cancer module; HRQoL EQ-5D Health Related Quality of Life EuroQol 5-Dimension; QLQ-ELD14 Quality of Life of Elderly Cancer Patients; EORTC-QLQ-C30 European Organization For Research and Treatment Quality of Life Questionnaire Core-30.

The Feasibility Pilot trial will recruit a target number of 50 consecutive patients. These patients will be only adult, either gender, with HR + HER2 negative mBC; ECOG- Performance Status (PS) ≤ 2; life expectancy > 12 weeks according to clinical judgment; candidate or in treatment with an oral agent including oral combination of endocrine therapies (ET) and CDK4/6 inhibitors, mono hormonal therapy (novel oral SERDs- selective estrogen receptor degrader, AIs-aromatase inhibitors, SERMs-selective estrogen receptor modulator, newer endocrine agents), combination ET with inhibitors of the PI3K (phosphatidylinositol 3-kinase (PI3K), -Akt (protein kinase B)-PTEN (Phosphatase and tensin homolog) pathway or of mechanistic target of rapamycin (mTOR), poly ADP ribose polymerase (PARP) inhibitors in case of BRCA carriers, oral chemotherapies (capecitabine, vinorelbine, cyclophosphamide). A summary of the pilot study design is shown in Figure 1.

## 3. Results

### 3.1. Survey and Focus Groups Results

The questionnaire revealed that the treatment process is often not clear and that information about the disease state is fragmented and poorly aq. The questionnaire confirmed that the lack of information, uncertainty, and indefinite timeframes can lead to anxiety, loss of control, low confidence, and depression, and is often followed by the need to seek information online or from friends and acquaintances. The majority of patients reported that printed information facilitated better discussions with healthcare providers, provided desired confirmation or answers, and occasionally prompted them to raise questions they hadn’t previously thought of. Furthermore, the results of the survey indicate a clear preference among respondents for the integration of digital tools, particularly mobile applications, into their healthcare experience, especially concerning communication with operators and information access, the possibility of consulting at any time information and educational material in support of self-management, and keeping a clinical diary shared with operators. Two focus groups were subsequently conducted by an experienced facilitator specializing in user experience on two separate occasions, with a total of 11 patients (of differing ages, social backgrounds, and educational qualifications). Objectives of the focus groups comprised the collection of patients’ experiences, as well as the identification of their primary needs, difficulties, and information gaps perceived during disease pathways. Table 2 shows some user stories from the 2 focus groups. Guided by a comprehensive analysis of patient needs identified through surveys, interviews, and existing literature, the decision was made to integrate a diverse set of functionalities into the app.

### 3.2. Analysis of the Clinical Process

**Figure 2:**
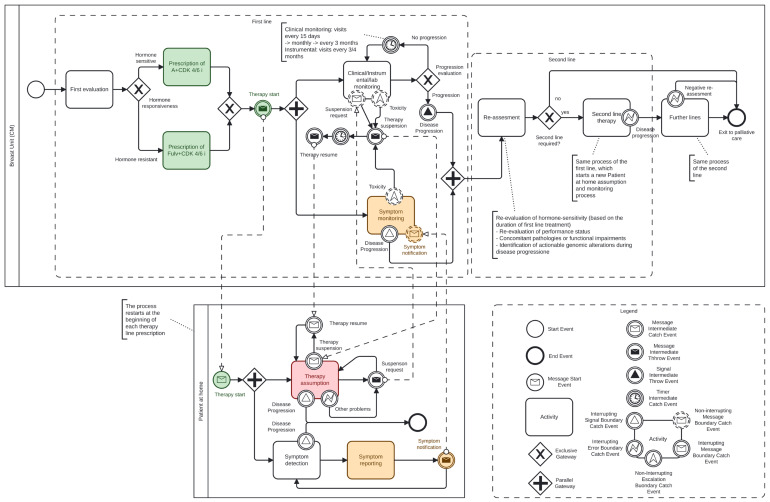
BPMN Diagram of the metastatic breast cancer pathway (see legend explanation in Section 3.2). The colored boxes show where the TREC_platform is applied.

The integration of TreC_Metha into the management of mBC patients addresses the complexity of metastatic courses, including a plethora of clinical, psychological, and social factors. A comprehensive analysis was conducted of the disease progression and the therapeutic interventions employed, with particular attention to the patient‘s expressed requirements. This analysis was undertaken within the context of HR + HER2-negative metastatic disease, which is the most prevalent subtype (65–70% of cases) and where many treatment plans provide for fully or partly oral therapy even for long periods of time. In this section, we present the treatment process for mBC, centered on the use of medications taken by the patient at home. The result of the analysis above is represented in the BPMN diagram shown in Figure 2, which illustrates the patient management process, including clinical evaluations, communication events, and decision points across different phases of care.

The use of a human-readable formal notation is pivotal for two main reasons: first, it facilitates consensus on process details among domain experts; second, it ensures an unambiguous representation of the process, thereby supporting the definition of application functionalities at each stage. The meanings of the graphical symbols used in the diagram are explained below (see also the legend box in Figure 2, which shows the correspondence between names and symbols):

Activity represents an activity carried out by an actor of the process, either the care team or the patient;

Exclusive gateway represents a decision point where only one path is chosen based on some condition (e.g, hormone therapy or not);

Parallel gateway splits the process into multiple parallel activities (e.g., Instrumental monitoring and symptom monitoring);

Start Event marks the beginning of a process. It triggers the workflow when a specific condition or signal occurs;

End Event indicates where a process ends. It signifies the completion of the workflow;

Message Start Event starts a process when a specific message is received from an external participant or system (e.g., the therapy starts);

Message Intermediate Throw Event sends a message to an external participant or system as part of the ongoing process (e.g., the patient asks for the suspension of the therapy);

Message Intermediate Catch Event waits for a specific message to be received during the process, which then allows the flow to continue (e.g., a request for therapy suspension);

Signal Intermediate Throw Event broadcasts a signal to any listening processes (e.g., a toxicity event occurred); unlike messages, signals are not targeted to a specific recipient;

Interrupting Signal Boundary Catch Event is attached to the boundary of an activity; if a signal is received (e.g., disease progression), it interrupts the activity and diverts the flow;

Interrupting Error Boundary Catch Event catches an error thrown by the activity it is attached to (e.g., an unexpected problem); when triggered, it interrupts the activity and moves to an error-handling flow;

Interrupting Message Boundary Catch Event interrupts the activity if a specific message is received (e.g., the suspension of therapy), redirecting the process flow to a different path;

Non-Interrupting Message Boundary Catch Event responds to a received message (e.g., the notification of a symptom) without interrupting the ongoing activity; the process continues along both the original and new flow paths;

Non-Interrupting Escalation Catch Event: catches an escalation signal (e.g., a toxicity) without stopping the activity; it allows parallel handling of escalation while the main activity continues;

Intermediate Timer Catch Event pauses the process flow until a specified time or duration elapses (e.g., “wait 1 week before resuming a therapy”).

The process is divided into two distinct pools. The upper pool (Breast Unit) is representative of the hospital setting, wherein healthcare professionals are responsible for the management of patients. The lower pool (Patient at home), on the other hand, represents the patient engaging in activities related to their own care within the domestic environment.

The colors assigned to some symbols in the diagram represent the points of the process in which the app provides specific supporting functionalities (see Section 3.3).

#### 3.2.1. Analysis of the Clinical Process in Hospital Setting Pool

In this subset of patients, the standard of care first line is the oral combination of endocrine therapies (ET) and CDK4/6 inhibitors, achieving a median PFS of about 25 months [47].

An initial assessment of tumor hormone responsiveness is necessary for deciding the first-line therapy, which assesses and determines two possible treatment paths (Hormone responsiveness diamond):

Hormone-sensitive patients receive a prescription for Aromatase Inhibitors (AI) + CDK 4/6 inhibitors.

Hormone-resistant patients (patients in progression of disease during adjuvant AI therapy for early breast cancer) are prescribed Fulvestrant (Fulv) + CDK 4/6 inhibitors.

The possibility exists for the dose to be reduced, the therapy to be delayed, or it to be stopped temporarily or indefinitely due to side effects, complications of the disease, or the need for additional treatments for the oncological disease (e.g., palliative radiotherapy or surgery) or for other diseases being treated concurrently. All options must be discussed candidly with patients to facilitate their capacity to reach a well-informed, shared decision.

For this, once therapy is initiated, two parallel activities are activated and executed by the care team:

1. Clinical, instrumental, and laboratory monitoring. In this activity, the oncologist team continuously controls the treatment course, which includes visits every 15 days initially, and then monthly, with monitoring of blood count, hepatic, renal function through regular blood tests, and psychological distress;
2. At the same time, symptom monitoring relies on the patient’s self-reporting to the care team of any symptoms that may arise following home therapy administration (reception of the message Symptoms notification from the Patient at home pool). Currently, the communication occurs by phone or by visiting the ER.

Furthermore, the medication prescription triggers the start of the process in the Patient at home pool.

Despite the improvements achieved, resistance to ET and CDK4/6is, as well as tumor progression, may be an occurrence following extended periods of disease control in which disease-related symptoms are observed to be non-existent or minimal. Hence, throughout the treatment, the process includes regular disease progression assessments by clinical, laboratory, and instrumental examinations (Progression evaluation diamond).

In the event of progression, a Disease progression signal event is triggered, causing the termination of activities in the First line group and the process in the Patient at home pool.

Subsequently, a reassessment is conducted, which may result in one of the following outcomes:

- Second-line ET (with or without other targeted agents), if appropriate;
- Cross to chemotherapy (or Antibody drug conjugates) regimen;
- Transition to exclusive palliative care if further treatment is not viable.

After progression to first-line ET-CDK4/6i combination, there exists a paucity of evidence to substantiate the optimal sequence of therapeutic interventions. The criteria currently in use pertain to the duration of first-line therapy, the disease burden, and the presence of any actionable alterations, as well as the symptomaticity of the disease.

It has been observed that a small subset of patients may no longer respond to ET after fast progression of disease to CDK4/6 inhibitors (primary resistance), despite the persistence of elevated ER or PR expression. This patient subgroup has been shown to demonstrate no significant benefits from endocrine therapy (ET) monotherapy, or from the continuation of cyclin-dependent kinase 4/6 inhibitors (CDK4/6is), or from the inhibition of alternative estrogen receptor (ER)-independent signal transduction pathways. In such cases, a more effective strategy may be chemotherapy, and eventually antibody–drug conjugates (ADCs). The identification of key actionable genomic alterations in HR+/HER2− mBC patients (somatic ESR1 or PI3K/AKT/mTOR mutations or germline mutational drivers such as BRCA1,2) could guide the selection of tailored treatment (oral SERDs, alpelisib, capivasertib, or PARP inhibitors) in subsequent lines of care. The identification of resistance drivers could facilitate the definition of potential therapeutic strategies that would overcome the problems of ET and/or CDK4/6i resistance.

Such strategies might encompass the following approaches:

1. The continuation of a CDK4/6i, in conjunction with the administration of an alternative ET or a re-challenge of the treatment with another CDK4/6i (median PFS 5–6 months) [48,49];
2. Targeting of an altered endocrine pathway, encompassing fulvestrant, novel oral SERDs and an array of other novel endocrine agents, including SERMs, SERCAs, CERANs and PROTACs among patients who had longer prior exposure to CDK4/6is (at least 12 months), especially ESR1-mutant (median PFS 8 months in this specific subset). Inhibiting alternative estrogen receptor-independent signaling pathways (such as the PI3K/AKT/PTEN pathway) by alpelisib [50] and capivasertib, achieving, respectively, a median PFS of 11 and 7 months [51];
3. Inhibiting pathways impacted by BRCA1,2 germline mutations (PARP inhibitors) by olaparib or talazoparib [52,53,54].

As the disease progresses, in fact, the lines of treatment (hormonal or chemotherapeutic) are utilized sequentially, resulting in a tendency for progressively diminishing PFS intervals. Progression pattern of the HR + HER2 negative mBC is heterogeneous and may be more or less rapid depending on a number of factors, including the biological aggressiveness of the disease itself, the extent of visceral involvement, the disease burden, and the patient’s general condition. The monitoring of disease evolution, in conjunction with clinical and instrumental re-evaluation, represents opportunities to evaluate the treatment outcomes in relation to patient side effects and general condition, as well as the psychosocial aspects reported by the patient. Each reassessment step must be discussed and shared with the patient, and any decision about subsequent treatment must be personalized, weighing benefits, goals, and side effects so that a collaborative and informed therapeutic choice can be made, considering the overall QoL.

This prescription-monitoring-assessment cycle repeats itself across subsequent therapy lines with each disease progression (represented for simplicity in the diagram by the Second line group and the Further line activity).

Additionally, the process accounts for temporary therapy suspensions, which may occur due to the following reasons:

- Toxicity related to the treatment, as detected through the two monitoring activities mentioned above;
- Patient requests for personal or medical reasons;
- Other clinical or logistical issues that may arise during therapy administration (e.g., occurrence of other morbid conditions related or unrelated to the underlying oncological pathology).

If the care team decides to suspend the therapy, the decision is notified to the patient (message Therapy suspension). However, the monitoring activities by the care team continue. Similarly, when the care team decides to resume the therapy, the patient is notified (message Therapy resume) and resumes taking the medication.

#### 3.2.2. Analysis of the Clinical Process in ‘Patient at Home’ Pool

The process in the “at home” pool begins with the patient receiving instructions to start therapy intake with the medication prescribed by the care team. The patient is responsible for two key activities:

- Taking the prescribed therapy as instructed by the care team;
- Monitoring and reporting symptoms to the care team that may indicate toxicity caused by the medication.

Additionally, during the treatment, the patient can request therapy suspension from the care team (message Suspension request) at any time for any reason (e.g., the need for a break or other issues). The care team may decide to approve the suspension and will inform the patient (message Therapy suspension), who then stops taking the medication for the agreed-upon period. After this period, the patient is notified by the care team to resume the therapy (message Therapy resume). During the suspension period, the patients continue to monitor their symptoms and inform the care team in case any significant symptoms occur.

The occurrence of disease progression (indicated by the reception of the Disease Progression signal event) results in the interruption of the patient’s therapy intake process for the corresponding treatment line, as well as symptom monitoring, while awaiting a new assessment and a decision on the next line of therapy. The latter will restart the process in the Patient at Home pool.

### 3.3. Technological Tools

The Trec_Metha project includes the development of an application that has been built upon the foundations laid by two previous projects in the cancer domain based on the TreC platform: Onco-TreC and TreC-Arianna.

Onco-TreC aimed to provide oncology patients treated with oral cancer therapies at home with an app-based electronic diary that allowed the oncologist to monitor the therapy and adverse events [55]. TreC-Arianna is a platform for managing the clinical pathway of early BC patients, from diagnosis through surgery and medical therapy to home treatment, physical rehabilitation, and follow-up, consisting of an app for patients and a dashboard for healthcare professionals. The platform provides contextual information to patients depending on the phase of the care pathway and allows the collection of different parameters to monitor the process [56].

The TrecMetha project combines the expertise, knowledge, and experience from the two projects and applies them to the management of patients with mBC cancer, in which it is imperative to undertake continuous monitoring of the treatment in order to ensure appropriate identification and management of any adverse effects or complications. Furthermore, ensuring that the correct information is provided to the patient at the optimum time is crucial to the effective running of such a program.

The required backend modifications include implementing a set of oral therapies for mBC, which can be prescribed by oncologists and displayed in the patient’s app, as well as defining a model for new self-reported data to enable patient monitoring at home. The platform provides functionalities that automatically adjust the configuration of the user’s app, contingent upon the phase of the patient’s treatment journey, including active treatment, paused periods, initial lines of treatment, and subsequent lines, discontinuation of the therapy or reduction in its dosage due to the occurrence of complications and so on. For this purpose, a specific service will be designed to visualize the BPMN process diagram in Figure 2 on the dashboard, allowing the healthcare team to click and select the currently active phase of the patient’s pathway. Based on this selection, the service configures the app content accordingly, including multimedia materials to be delivered and the set of parameters in the data collection forms. This feature ensures real-time monitoring and fosters a more comprehensive approach to therapeutic management.

These functionalities represent a significant innovation in relation to existing systems in terms of patient empowerment. They enhance patient awareness and active involvement in disease management, which has the potential to provide substantial additional benefits to treatment outcomes. Additionally, the platform’s dashboard enables healthcare professionals to oversee and adjust the status of care process activities by monitoring parameters and directly communicating with patients, thereby facilitating timely decision-making.

### 3.4. Application Structure and Functionalities

Based on the analysis of the care process and the needs of patients and healthcare professionals, the TreC_Metha platform implements the following functionalities, summarized in Table 3, structured across two main access points: a dashboard for healthcare professionals, serving as their interface to monitor patients, manage treatments, and provide guidance, and a patient app.

#### 3.4.1. Healthcare Professional Dashboard

Patient Registration and Access Management: To be compliant with security standards, patient access to the application is managed through a dedicated registration process that includes the creation of credentials with multi-factor authentication. The credentials are created by an authorized healthcare professional during the initial evaluation phase when the patient is enrolled in the pathway.

Event and Intervention Documentation: The system is able to systematically record all the sequences of events and all interventions undertaken by healthcare professionals. This is carried out via a web-based dashboard, ensuring a structured and traceable care process.

Configurable Parameter Acquisition: The system allows the configuration of specific mBC-related parameters to be entered in the app by the healthcare professional (patient status, concomitant pathologies, contingent information upon the phase of the patient’s treatment journey), alongside parameters common to all patients. This functionality was designed to support the activities related to the reporting of symptoms self-reported by the patient, colored in yellow in the BPMN diagram.

Recording of relevant information: Through the web-based dashboard the healthcare personnel is able to report and store in the system database, clinical information to be visualized in the patient’s app: (i) the therapy the patient is following, which may be modified during the course of the study; (ii) practical suggestions and advices for lifestyle improvement and patient empowerment delivered via messages visualized in the app. This functionality was designed to support the activities related to the therapy prescription and assumption, colored in green and in light red in the BPMN diagram.

Activity Setup for Patient Engagement: To enhance treatment adherence and patient engagement, the system enables the setup of specific health-related activities requiring the patient to enter data, supported by in-app reminders. The collected data are stored in the system’s database and displayed in a dedicated telemonitoring section of the dashboard, providing clinicians with a comprehensive view of the patient’s clinical progress. This functionality was designed to support the activities related to the monitoring of the medication, colored in yellow and in light red in the BPMN diagram.

Communication channels: The platform integrates both asynchronous (chat, similar to WhatsApp) and synchronous communication channels (teleconference). This functionality will facilitate direct communication between patients and healthcare providers, thereby enhancing accessibility to healthcare services.

Therapy Reminder System: Through the patient’s app, the system delivers reminders and notifications for medication intake, promotion of specific wellness-related behavior, rehabilitation tutorials sent via images, multimedia content, and educational material. The dashboard should allow the monitoring of a patient’s engagement with these resources. This functionality was designed to support the assumption of the therapy by the patient (red colored activity in the diagram) to improve their adherence.

Automated Report Generation: The dashboard supports the automated generation of routine reports based on patient-entered data. These reports are structured in a consolidated document, thereby providing valuable insights for long-term monitoring and clinical decision-making.

The synergy of these components is set to enhance the efficacy of the care pathway, with an attendant improvement in treatment adherence, patient engagement, and, ultimately, enhanced quality of care.

#### 3.4.2. Patient’s App

Synchronous and asynchronous communication channel: The patient app includes a video-chat feature that enables real-time (synchronous) communication between the patient and the healthcare professional, who accesses the corresponding video-chat tool through their dashboard. This function is particularly useful for remote consultations, enabling direct discussions about treatment plans, symptoms, or concerns. To prevent unnecessary interruptions, video consultations are initiated exclusively by the physician and are reserved exclusively for cases of a particularly complex nature, which require immediate medical attention. The app also offers a chat functionality linked to the corresponding chat channel in the professional’s dashboard, enabling text-based communication with the healthcare team and facilitating the exchange of documents and images.

Personal diary: Patients have access to a personal diary where they can track key health parameters. This includes standard parameters applicable to all users, as well as specific metrics linked to tasks assigned by healthcare providers. The diary also integrates reminders, ensuring that patients remain aware of their scheduled activities and health-related tasks, fostering a more proactive approach to disease management. This functionality was designed to support symptom reporting and monitoring activities, colored in yellow in the diagram.

Drug diary: The application features a drug diary function that allows patients to record their prescribed medications and therapies, as documented by their physician via the clinical dashboard. Additionally, patients can independently record any over-the-counter medications or alternative therapies they use, particularly those taken to manage side effects. This functionality provides both patients and healthcare providers with a comprehensive view of medication adherence and potential interactions, ultimately resulting in improved overall management of treatment.

Reminder and notifications: To facilitate adherence to the care plan, the application automatically generates notifications at regular intervals, reminding patients to complete scheduled activities. These activities include medical appointments, pending health actions, and medication intake, as well as recommendations for rehabilitation exercises and lifestyle modifications. The notifications are designed to ensure that patients continue treatment and lifestyle guidance even when the application is closed. This functionality was designed to support the assumption of the therapy at home (red colored activity in the diagram).

Delivery of questionnaires: Questionnaires will be made available via the application for assessing the feasibility of the system in terms of patient-reported experience (PREMS), patient-reported outcomes (PROMS), and clinicians’ expectations. This information will be timely presented to clinical professionals through the web dashboard and will support decision-making during the treatment process.

**Figure 3:**
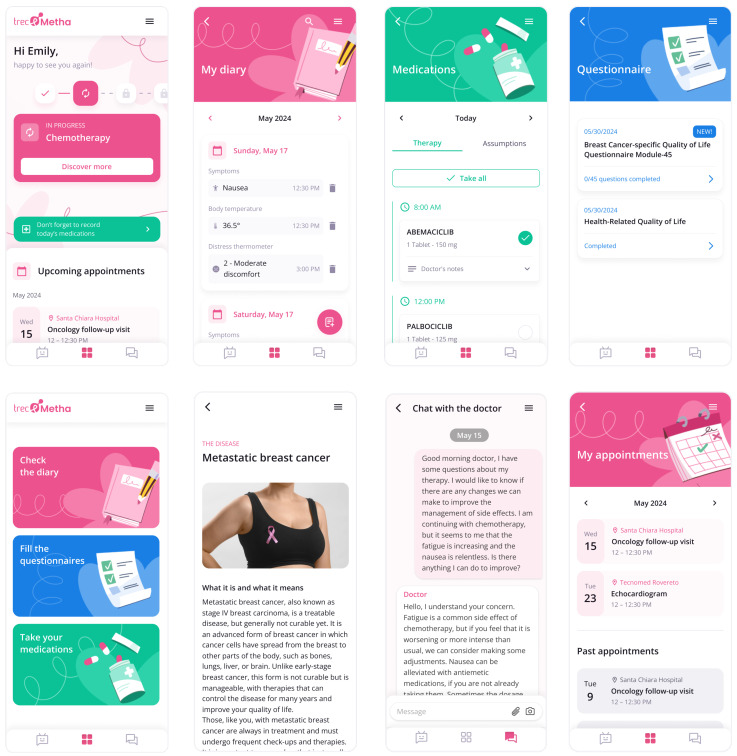
Screenshots of the Trec_Metha mobile application showing the therapy calendar (**upper left**), the daily schedule upper (**upper middle**), the chat with the clinician (**upper right**), drug intake (**bottom left**), the contextual information content (**bottom in the middle**), and dispensing questionnaires (**bottom right**).

Figure 3 shows the screenshots of the main functionalities of the patient’s app.

## 4. Discussion

Recent advances in hormonal therapies, targeted agents, and antibody–drug conjugates (ADCs) have significantly improved the prognosis of HR+HER2-negative metastatic breast cancer (mBC). Luminal mBC is characterized by a protracted therapeutic continuum, encompassing extended periods of disease control and intermittent relapses that necessitate modification of the therapeutic strategy. Patients diagnosed with mBC face a number of specific barriers when they are challenged by a life-limiting prognosis, including a variety of complexities such as uncertainty, functional impairment, changes in social and occupational roles, fear of death and suffering, concern for family members, and communication with healthcare professionals. In addition, patients with metastatic disease often experience significant disease-related symptoms and treatment-related adverse events. It is therefore imperative that enhancements in disease control and OS are accompanied by maintaining quality of life (QoL) [57]. In order to facilitate effective shared decision-making, medical professionals must provide patients with clear, comprehensive information regarding the benefits, risks, and uncertainties associated with treatment options. Moreover, there is a requirement for a substantial effort to be made in terms of communication, ensuring semantic adequacy and an understanding of the perspective of the patient, in the context of shared decision-making [58].

However, in spite of the provision of adequate information and a strong patient-care team relationship, many patients turn to unverified online sources [59]. Therefore, it is crucial that they are directed to reliable, accredited information recommended by their healthcare team. Indeed, it is important that patients are not passive recipients of care; rather, they are active managers of their own health. The collection of the patient’s experience throughout the cancer care pathway plays an important role in improving communication, adapting and/or improving clinical and supportive care, increasing psycho-physical well-being, ensuring long-term care and health management, and ultimately improving QoL [60]. In addition, the implementation of QoL assessments into clinical practice for BC treatment has the potential to benefit patients to a significant degree [61,62,63].

Technologies offer an efficient and accurate means of implementing QoL assessments. Access to digital systems and technological devices has been shown to engender positive change in health and well-being [64]. Recent studies have demonstrated the efficacy of telemedicine in delivering a versatile and adaptable framework for the provision of support, the monitoring of PROMs, and the promotion of seamless continuity of care across both intra- and extra-hospital settings [65,66,67,68,69]. eHealth, in all its complexity, can be considered a valuable resource in mBC, offering a wide range of applications. These include online platforms that facilitate the provision of important clinical information, management of therapy adherence, continuous educational support, and promotion of self-management [70]. These dedicated tools and solutions are designed to address specific challenges related to improving long-term outcomes and QoL, thereby enhancing the overall well-being of patients. In this article, we described the design of the platform TreCMetha, aimed at supporting and monitoring patients with HR + HER2-mBC, and how it will be integrated throughout the care process.

The meticulous and comprehensive healthcare pathway analysis conducted as a preliminary step was complemented by a review of the role of digital tools in BC management. This analysis demonstrated that the implementation of digital health platforms significantly empowers patients and promotes treatment adherence, and facilitates more effective self-management practices. The user centric design approach employed in this study, which integrated input from all stakeholders (software developers, healthcare team and patients) confirmed user demand for several functionalities present in existing platforms, such as: evidence-based digital information, treatment phase, medication tracking and appointment scheduling caps, question-and-answer (Q and A) function. Additionally, our process revealed significant unmet needs that guide us to optimize the possibility for the professionals to access data entered by users simultaneously. This process led us to develop the TreCMetha platform, consisting of a web dashboard for healthcare professionals and a mobile app for patients designed in strict collaboration with the final users. A significant innovation inherent to this TrecMetha project pertains to the development and implementation of a tool that automatically adjusts the app’s configuration based on the patient’s treatment phase. This includes active treatment, pauses, initial and subsequent lines of therapy, treatment discontinuation, and dosage reductions due to complications.

## 5. Conclusions

In this paper, we presented the design and development of TreC_Metha, a digital platform developed to support patients with luminal HR+/HER2-negative metastatic breast cancer. The platform’s functionalities were designed to facilitate information exchange between patients and healthcare teams, offer guidance on both organizational and clinical aspects of care, and enhance patient awareness and engagement throughout their care journey. To our knowledge, no such comprehensive instrument exists that would enable involvement and active participation in the mBC care process for both a multidisciplinary care team of professionals and the patient.

In the next phases of the project, a group of HR + HER2-mBC patients will participate in usability testing of the platform using an interactive wireframe, which is a graphical prototype that provides a realistic representation of its visual structure and functionality. Using a fully functioning application’s prototype installed on their mobile devices. Following the collection, integration, and implementation of feedback from the initial prototype testing, a pilot study will be conducted to evaluate the acceptability, usability, and potential impact of TreC_Metha on patients’ QoL.

The platform could be scaled to benefit a broader patient population beyond the pilot scope and the mBC domain, thanks to TreC_Metha‘s inherently modular and extensible design, which allows for rapid adaptation to other oncology domains. This scalability is supported by a set of well-defined technical and organizational mechanisms:

Redefinition of the Monitored Data Model

The platform supports flexible extension of its data collection layer, enabling the definition and integration of new sets of monitored parameters tailored to specific cancer types (e.g., lung, colorectal). The data model can evolve over time without affecting the core structure of stored data and supports mapping to standard communication formats for interoperability, such as FHIR.

Redesign of the Care Process in BPMN

Each cancer type is supported by a dedicated care pathway, modeled using BPMN (Business Process Model and Notation). This formal representation acts as a runtime reference for the platform, enabling the contextual delivery of information, reminders, and alerts aligned with the clinical workflow of the selected oncology domain. As such, extending the platform to additional domains simply requires adapting or redesigning the relevant care pathway.

Definition of new informative educational content

For each new use case, pathology-specific content—such as patient education materials and recommended actions delivered at defined steps of the care pathway—is formalized and integrated into the platform’s knowledge base. This ensures consistent support across multiple conditions while maintaining the unique characteristics of each pathology.

Healthcare Professional Assignment and Segregation

The platform supports role-based access control and allows for the segregation of healthcare teams along various dimensions—such as clinical specialty, pathology-specific multidisciplinary teams, or hospital wards. Professionals are enrolled and assigned to distinct organizational units, each managing its own patient cohort, ensuring secure, focused, and domain-specific care delivery.

Multi-Provider Strong Authentication

To support secure access for both patients and professionals, the platform integrates multiple strong authentication methods, including One-Time Password (OTP), SPID (Italy’s Public Digital Identity System), and OAuth-based login via providers like Google. This ensures compliance with GDPR and national eHealth regulations.

These mechanisms ensure that the platform can be securely and efficiently scaled to support a growing number of patients and healthcare teams across diverse clinical domains.

These functionalities represent a substantial innovation in relation to contemporary systems, with regard to the empowerment of patients, aiming to increase their awareness and active involvement in the overall course management of their disease.

## Figures and Tables

**Table 2:** Focus group verbatim summary.

	Verbatim	Emerging Theme
#1	I need information about bureaucratic aspects too, like illness and work…	Digital tools for non-clinical informational needs
#2	Having a clear list of direct contacts, I can reach out to…	Digital tools for healthcare contacts
#3	I used a paper calendar where I wrote everything: appointments, exams, therapies…	Digital tools for self-management
#4	Getting info about my clinical pathway is fine, unless it changes and I’m not informed… it’s hard to have a clear plan from the start.	Digital tools for non-clinical informational needs and clinical updates
#5	I used Google Calendar to remind myself to take home medications…	Digital tools for self-management
#6	In the first days after diagnosis, there’s a need to know everything, so we look for information online…	Digital tools for clinical and non-clinical informational needs
#7	A chat with case managers wouldn’t work because they’re already overloaded, and I don’t know how it would be managed…	Digital tools for direct communication
#8	We need not only clinical information, but especially the kind doctors consider secondary…	Digital tools for clinical and non-clinical informational needs (Importance of practical/lifestyle information)
#9	The app could be a tool where I find information that I know is true, consistent, and science-based, without getting mixed answers…	Digital tools for the reliability and consistency of information
#10	If I enter data in a diary and receive feedback, that would be very useful…	Digital tools for recording personal narratives
#11	Yes, I would use the diary as a reminder before the oncologist visit…	Digital tools for recording personal narratives
#12	The informational content should be the top priority…	Digital tools for clinical and non-clinical informational needs
#13	I didn’t use a personal diary, but I would use an app to note my emotions. I wouldn’t want a chatbot telling me how I should feel…	Digital tools for recording personal narratives

**Table 3:** Definition of platform functionalities.

Level	Functionality	Description
Dashboard	Activation of credentials for the patient to access the application	- To register the patient; - To create the patient’s credentials for the multifactor authentication; - To activate the app via the healthcare provider’s web dashboard.
	Configuration of parameters that can be entered in the app	- To configure the acquisition of specific parameters in the patient’s app, in addition to those common to all patients; - To set up activities that require the collection of parameters, which can be linked to reminders in the app for the patient.
	Display of parameters (dashboard)	- To retrieve from the system’s database the parameters entered by patients and visualize them in the telemonitoring section of the dashboard.
	Activation and use of a chat between doctor/healthcare personnel and patient	- To provide an asynchronous communication channel (WhatsApp-like) between the doctor/healthcare professional and the patient through the app.
	Therapy reminder setting	- To send notifications of therapies prescribed by the physician; - To define reminders to the patient to take certain medications or engage in certain wellness behaviours.
	Configuration of routine reports	- To configure the generation of regular reports on the data entered by the patient at a specified frequency; - To consolidate the acquired data in a single document.
App	Implementation of a video chat between doctor/healthcare personnel and patient	- To provide a synchronous communication channel (video + audio) for remote discussion between a doctor/healthcare professional and a patient; - To avoid unnecessary disturbances, the video chat can only be activated by the doctor in the dashboard for particularly complex cases.
	Personal observations diary (for patient)	- To view the set of parameters defined for all patients, as well as those related to activated tasks, including any associated reminders.
	Activation of the drug diary	- To view and log the intake of medications and therapies prescribed by the physician via the clinical dashboard; - To enter and track any non-prescribed drugs and therapies taken independently or due to side effects.
	Generation of reminders	- To receive push notifications for appointments, pending tasks, medication intake, and more, even when the app is not active.

## Data Availability

The original contributions presented in this study are included in the article. Further inquiries can be directed to the corresponding author.

## Abbreviations

The following abbreviations are used in this manuscript:

ADCs	Antibody–Drug Conjugates
BC	Breast Cancer
BPMN	Business Process Model and Notation
ET	Endocrine Therapies
GDPR	General Data Protection Regulation
HR	Hormonal Receptor
mBC	Metastatic Breast Cancer
mTOR	Mechanistic Target of Rapamycin
OMG	Object Management Group
PARP	Poly ADP Ribose Polymerase
PFS	Progression Free Survival
OS	Overall Survival
PGHD	Patient-Generated Health Data
PREMS	Patient-Reported Experience
PROMS	Patient-Reported Outcomes
QoL	Quality Of Life
